# Stem cell-derived gametes: what to expect when expecting their clinical introduction

**DOI:** 10.1093/humrep/deaf123

**Published:** 2025-07-02

**Authors:** Ilse J de Bruin, Merel M Spaander, Simone Harmsen, Rosanne Edelenbosch, M Corrette Ploem, Nina Dartée, Madalena Cardoso Vaz Santos, Mathangi Lakshmipathi, Callista L Mulder, Ans M M van Pelt, Willy M Baarends, Susana M Chuva de Sousa Lopes, Guido M W R de Wert, Seppe Segers, Geert Hamer, Ana M Pereira Daoud

**Affiliations:** Department of Developmental Biology, Erasmus MC, Rotterdam, The Netherlands; Department of Health Law, University of Amsterdam, Amsterdam, The Netherlands; Department of Ethics, Law & Humanities, Amsterdam University Medical Center, Amsterdam, The Netherlands; Rathenau Institute, The Hague, The Netherlands; Rathenau Institute, The Hague, The Netherlands; Department of Health Law, University of Amsterdam, Amsterdam, The Netherlands; Department of Ethics, Law & Humanities, Amsterdam University Medical Center, Amsterdam, The Netherlands; Department of Developmental Biology, Erasmus MC, Rotterdam, The Netherlands; Reproductive Biology Laboratory, Center for Reproductive Medicine, Amsterdam UMC, University of Amsterdam, Amsterdam, The Netherlands; Amsterdam Reproduction and Development Research Institute, Amsterdam, The Netherlands; Reproductive Biology Laboratory, Center for Reproductive Medicine, Amsterdam UMC, University of Amsterdam, Amsterdam, The Netherlands; Amsterdam Reproduction and Development Research Institute, Amsterdam, The Netherlands; Reproductive Biology Laboratory, Center for Reproductive Medicine, Amsterdam UMC, University of Amsterdam, Amsterdam, The Netherlands; Amsterdam Reproduction and Development Research Institute, Amsterdam, The Netherlands; Reproductive Biology Laboratory, Center for Reproductive Medicine, Amsterdam UMC, University of Amsterdam, Amsterdam, The Netherlands; Amsterdam Reproduction and Development Research Institute, Amsterdam, The Netherlands; Department of Developmental Biology, Erasmus MC, Rotterdam, The Netherlands; Department of Anatomy and Embryology, Leiden University Medical Center, Leiden, The Netherlands; The Novo Nordisk Foundation Center for Stem Cell Medicine (reNEW), Leiden University Medical Center, Leiden, The Netherlands; Ghent-Fertility and Stem Cell Team (G-FAST), Department of Reproductive Medicine, Ghent University Hospital, Ghent, Belgium; Department of Health, Ethics and Society, Maastricht University, Maastricht, The Netherlands; Research Institute for Oncology and Reproduction (GROW), Maastricht University, Maastricht, The Netherlands; Care and Public Health Research Institute (CAPHRI), Maastricht University, Maastricht, The Netherlands; Department of Philosophy and Moral Sciences, Bioethics Institute Ghent, Ghent University, Ghent, Belgium; Reproductive Biology Laboratory, Center for Reproductive Medicine, Amsterdam UMC, University of Amsterdam, Amsterdam, The Netherlands; Amsterdam Reproduction and Development Research Institute, Amsterdam, The Netherlands; Department of Anatomy and Embryology, Leiden University Medical Center, Leiden, The Netherlands; Department of Medical Ethics and Health Law, Leiden University Medical Center, Leiden, The Netherlands

**Keywords:** stem cell-derived gametes, *in vitro* gametogenesis, human, induced pluripotent stem cells, clinical application, assisted reproductive technologies, ethics, law, society

## Abstract

Stem cell-derived (SCD)-gametes derived from induced or autologous (i.e. patient-specific) cells may help mitigate human fertility problems caused by physiological or social factors in the (near) future. While this technology is still in its infancy, recent advancements with SCD-gametes generated from mouse pluripotent stem cells have led some researchers to expect—and investors to anticipate—the clinical introduction of human gametes derived from induced pluripotent stem cells (iPSCD-gametes) within two decades. However, it remains to be investigated how realistic these expectations are, and how they would balance against careful consideration of technical, ethical, legal, and societal aspects, including—but not limited to—safety and effectiveness. This mini-review aims to encourage that investigation by providing a brief overview of the state-of-the-art and highlighting the breadth of issues involved in the potential clinical introduction of human iPSCD-gametes. These issues emerge before (Stage 1), during (Stage 2), and after (Stage 3) clinical trials, and are discussed in that order. Issues discussed in the context of Stage 1 suggest that gathering the evidence required to preclinically assess the safety of human iPSCD-gametes will be time-consuming and require parallel experiments with sensitive research materials. Issues discussed in the context of Stage 2 suggest that it might take several years for human iPSCD-gametes to transition through distinct clinical trial phases, and that inevitable (and unforeseeable) variations in the quality of human iPSCD-gametes are likely to further slow this down. Finally, issues discussed in the context of Stage 3 suggest that offering human iPSCD-gametes clinically will require addressing questions of accountability and monitoring, some of which might be difficult to formalize by law. Combined, these findings suggest that a responsible clinical introduction of human iPSCD-gametes may take considerably longer than expected, underscoring the importance of transdisciplinary collaborations with a broad range of stakeholders to make well-informed and well-considered choices about their development and application.

## Graphical Abstract

**Figure:**
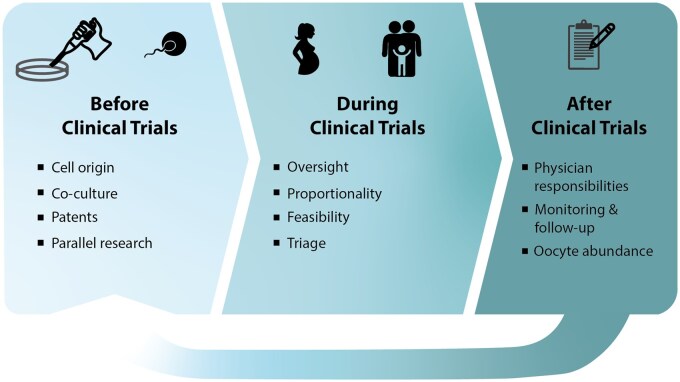
Considerations for clinical application of *in vitro*-derived gametes.

## Introduction

According to the latest estimate of the World Health Organization (WHO), at least ‘one in six people of reproductive age worldwide experience infertility’ (WHO, 2023). This is probably a conservative estimate due to inaccuracies in measurements across national surveys, the stigma and taboo associated with the handicap, and the fact that infertility is still broadly defined as having strictly physiological, rather than situational, causes (OHCHR, 2023). Physiological infertility refers to people who cannot have genetically related offspring due to biological factors, such as testicular or ovarian disorders. By contrast, situational infertility refers to people who cannot have genetically related offspring due to legal, social, or cultural factors, such as homosexuality or involuntarily sterilization. Concepts of infertility are therefore not static and can overlap, in part because emerging ARTs continue to open new horizons for genetic parentage.

Recently developed techniques to generate egg and spermatid cells from mouse pluripotent stem cells (PSCs), referred to as stem cell-derived (SCD)-gametes, could in the future help mitigate fertility problems caused by physiological or social factors when applied to humans. When used in research, for example, SCD-gametes could help improve and expand the scientific understanding of human gametogenesis and reproduction, ultimately contributing to developing new clinical therapies. Still, it is the application of SCD-gametes for human reproduction that speaks the most to the imagination. Scientists anticipate the development of successful differentiation protocols for clinical introduction, with some estimating that could become reality within one to two decades (Cyranoski *et al.*, 2023; National Academies of Sciences, Engineering, and Medicine, 2023). Meanwhile, the field is beginning to catch the attention of venture capitalists, some of whom have already expressed hopes about ‘translating this technology to humans [and making] it a safe and accessible reproductive treatment’ (Conception.bio, 2023). It remains to be investigated how realistic these anticipations are, and how they balance against a careful consideration of ethical, legal, and societal concerns, including—but not limited to—the safety and effectiveness of the technology.

At present, the generation of human SCD-gametes remains in its infancy (Robinson *et al.*, 2023; Wu *et al.*, 2023). Despite proof-of-concept in mice (Hayashi *et al.*, 2011, 2017; Hikabe *et al.*, 2016), few studies have so far been directed at generating SCD-gametes from human embryonic stem cells (hESCs), and even fewer at generating them from human-induced pluripotent stem cells (hiPSCs). This distinction in cellular origin matters because hESCs are derived from the inner cell mass of human embryos (Thomson *et al.*, 1998), which is not only an invasive and (ethically) sensitive procedure (Rugg-Gunn *et al.*, 2023), but one that would make the embryo the genetic parent of the offspring generated from ESC-derived (ESCD)-gametes (Mertes and Pennings, 2008; Watt, 2014). By contrast, hiPSCs are autologous: they are derived from the reprogramming of adult somatic cells (Takahashi *et al.*, 2007; Park *et al.*, 2008). This means that human iPSC-derived (iPSCD)-gametes could be generated from less to non-invasive biopsies (e.g. skin, blood, or urine) of aspiring parents, establishing an immediate genetic link between them and any resulting offspring.

The reproductive use of SCD-gametes has attracted considerable interest over the past decade. On the one hand, the technology raises controversy about the many avenues it opens, including non-traditional family forms (such as solo or multiplex parenthood; Palacios-González *et al.*, 2014; Suter, 2016), eugenics (Guimarães da Fonseca *et al.*, 2014; Mertes, 2014a; Siegel, 2014; Sparrow, 2014), cloning (Segers *et al.*, 2019), gene editing (de Wert *et al.*, 2018), and possibilities for unwitting parenthood (Smajdor and Cutas, 2014). On the other hand, it raises hope about promoting inclusion and equity in human reproduction (Testa and Harris, 2005; Bredenoord and Hyun, 2017; Le Goff *et al.*, 2024), especially for those currently incapable of using their own gametes for conceiving genetically related children. Although the moral importance of genetic-centered parenthood can be questioned (Cutas *et al.*, 2014; Mertes, 2014a), it certainly is a major force driving the development of iPSCD-gametes (Hendriks *et al.*, 2015). To help set the stage for their responsible application, this mini-review summarizes the state-of-the-art in iPSCD-gametes and discusses immediate and immanent ethical, legal, and societal issues arising from (specific stages in) their potential clinical introduction. While scope constraints prevent this discussion from being exhaustive, it may help orient further reflection on the therapeutic potential of iPSCD-gametes.

## The state-of-the-art in iPSCD-gametogenesis

**Figure 1.**
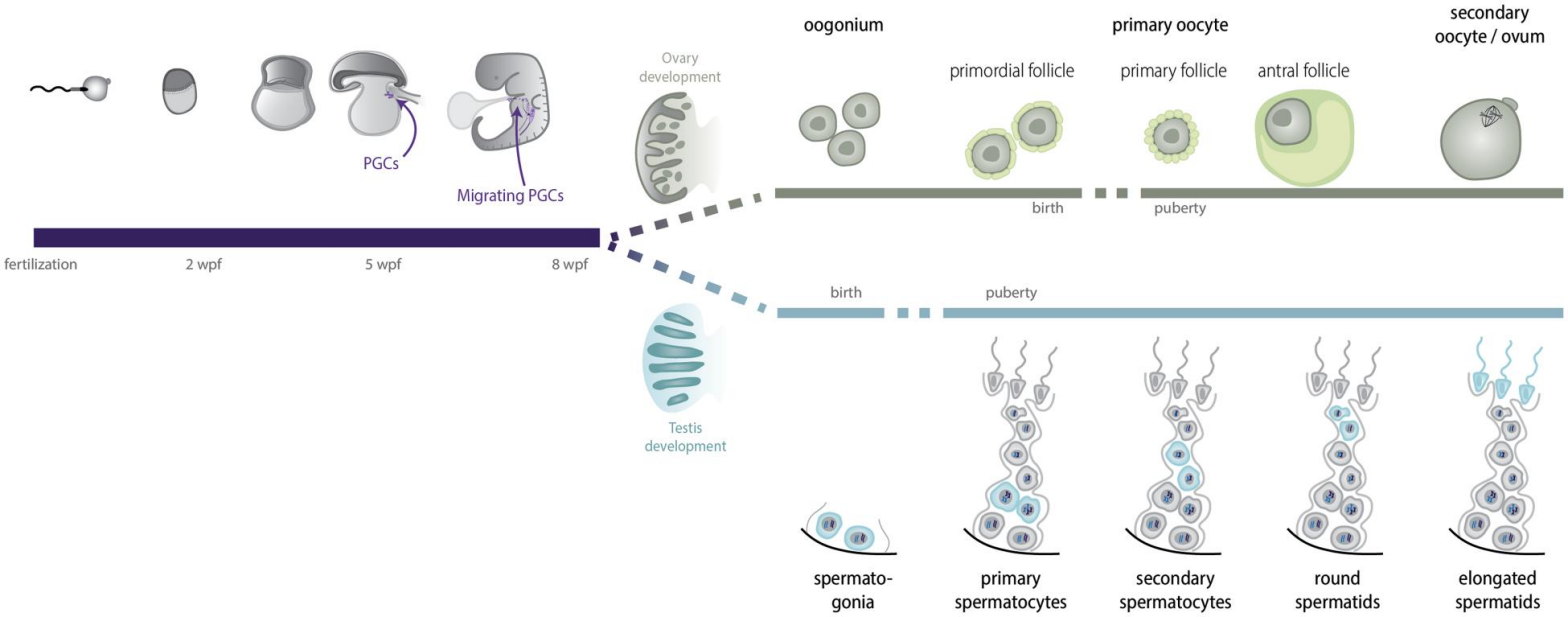
**Schematic overview of human gametogenesis**. Timeline showing *in vivo* development, starting from fertilization. Time shown in weeks post-fertilization (wpf). The upper panel shows the development within the ovary, the lower panel shows development within the testis.

In mammals, gametogenesis begins with the specification of primordial germ cells (PGCs) during early embryogenesis. After initial specification and proliferation, PGCs migrate to the genital ridges, where they are primed for sex-specific germ cell differentiation into the precursors of either egg cells (oogonia) or sperm cells (spermatogonia). Sex-specific differentiation continues; in oogenesis, by first initiating meiosis and then pausing until puberty or, in spermatogenesis, by first pausing and continuing from puberty onward (reviewed in Saitou and Hayashi, 2021) (Fig. 1).

**Figure 2.**
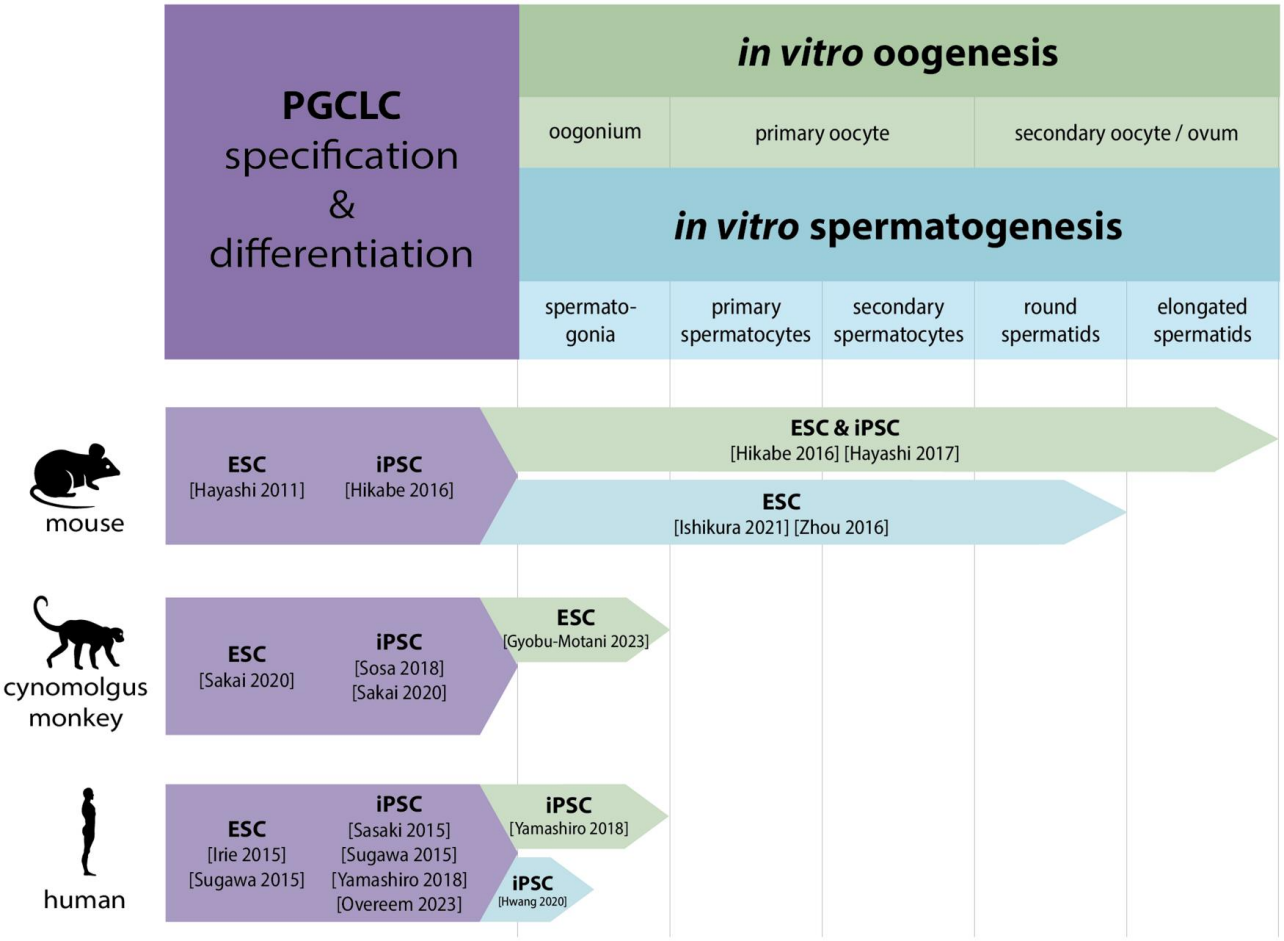
**Overview of recent progress in the field of *in vitro* gametogenesis in various species**. Each box shows the origin of the cells used (embryonic stem cells (ESCs) or induced pluripotent stem cells (iPSCs)) and the reference(s) where the protocol is described. Progress in primordial germ cell-like cell (PGCLC) derivation is shown in purple on the left, further progress in *in vitro* oogenesis is shown in green, while further progress in *in vitro* spermatogenesis is shown in blue. All references can be found in the reference list.

The creation of SCD-gametes would theoretically consist of similarly distinct steps (Fig. 2). Full *in vitro* protocols would begin with differentiating PSCs into PGC-like cells (PGCLCs), which could then be primed into either SCD-oocytes or SCD-spermatids through laboratory co-culturing techniques.

### From PSCs to PGCLCs

PGCLCs have been differentiated from the PSCs of several mammalian species, including rodent (Hayashi *et al.*, 2011; Oikawa *et al.*, 2022), non-human primate (Sosa *et al.*, 2018; Sakai *et al.*, 2020), and human (Irie *et al.*, 2015; Sasaki *et al.*, 2015; Sugawa *et al.*, 2015; Overeem *et al.*, 2023) (Fig. 2). As constraints on human embryo research limit studying human gametogenesis, mammalian animal models give useful insights. However, known and unknown differences between (mammalian) species call for more research to elucidate them, as the same protocols for *in vitro* gametogenesis (IVG) cannot be translated to other species without modifications. For instance, whereas mouse PGCLCs resemble a peri- or post-migratory state, expressing more mature PGC genes like *Ddx4/Vasa* and *Dazl* (Hayashi *et al.*, 2011), primate PGCLCs retain a pre-migratory (or immature) state (Sugawa *et al.*, 2015; Irie *et al.*, 2023; Overeem *et al.*, 2023), making them less suitable for co-cultures with fetal somatic gonadal cells at this moment. Hence, human PGCLCs require an intermediate maturation step, toward which approaches have been made (Irie *et al.*, 2023; Murase *et al.*, 2024). Another difference between mice and humans is that the PSCs are typically maintained in different pluripotency states (Tesar *et al.*, 2007; Nakamura *et al.*, 2016). Mouse PSCs have a naïve pluripotent state, with the ability to form both embryonic and extraembryonic tissues, and XX cells typically contain two active X chromosomes. On the other hand, human PSCs have a primed pluripotent state, harboring a certain epiblast lineage priming and XX cells having one active and one inactive X chromosome (Nichols and Smith, 2009). Since the starting pluripotency states differ, this might also affect the differentiation potential of the cells, although this remains to be thoroughly elucidated.

At present, various technical issues persist in differentiating human PSCs into PGCLCs. For example, the prolonged culture of existing PSC-lines could lead to mutations in the genome and epigenetic aberrations that lead to abnormal differentiation potential, many of which are still insufficiently understood (Choi *et al.*, 2017; Bar and Benvenisty, 2019; Poetsch *et al.*, 2022). These can have deleterious health effects in the future offspring. For this reason, PGCLCs intended for reproduction may need to be differentiated from newly derived PSC-lines with low passage numbers.

### From PGCLCs toward female gametogenesis

PGCLCs from various species have been differentiated and properly characterized, but further maturation toward oocytes has proven to be troublesome for some of them, highlighting differences between mammalian species (Fig. 1). In mouse, oogenesis has been fully recapitulated *in vitro* resulting in fertile offspring, albeit at low-efficiency rates and occasionally demonstrating abnormal development (Hikabe *et al.*, 2016; Hayashi *et al.*, 2017). Moreover, mouse SCD-oocytes have also been generated from chromosomally male PSCs (Murakami *et al.*, 2023), although precarious gene editing was necessary for XY to XX conversion. At present, the application of this procedure remains notably inefficient and raises significant safety concerns associated with the use of the missegregation agent, which could result in further unintended aneuploidies.

By contrast, oogenesis in primates has not yet been recapitulated fully *in vitro*. Research with non-human primates has shown that co-culturing cynomolgus monkey PGCLCs with embryonic day 12.5 (E12.5) mouse fetal ovarian somatic cells (FOSCs) leads to differentiation of meiotic oocytes that arrest at the zygotene stage (Gyobu-Motani *et al.*, 2023). Research with human PGCLCs is scarcer but their co-culture with E12.5 mouse FOSCs has been shown to result into oogonia-like cells through *in vitro* maturation (Yamashiro *et al.*, 2018). Presumably due to differences between mouse and human oogenesis, such as (a)synchronous development of PGCs, these human PGCLCs took considerably longer to mature than their *in vivo* counterparts would have had and did not initiate meiosis. To bypass these and other translational challenges between species, the ideal scenario would be co-culturing human PGCLCs with either *in vitro-*derived human FOSC-like cells (as has been done in mice, Yoshino *et al.*, 2021), or human fetal ovaries (Yang *et al.,* 2022; Overeem *et al.*, 2023).

### From PGCLCs toward male gametogenesis

Similar approaches are being developed for mouse SCD-spermatids, by co-culturing PGCLCs with mouse fetal testis somatic cells (FTSCs) and transplanting them into germ cell-deprived mouse testes. This aided the PGCLCs to differentiate into functional spermatid-like cells (Ishikura *et al.*, 2021). *In vitro* differentiation from mouse PGCLCs into haploid spermatid-like cells that resulted in offspring after ICSI has also been reported (Zhou *et al.*, 2016). Full *in vitro* protocols starting with human PSCs and co-cultures with mouse FTSCs reported obtaining differentiation into prospermatogonia (Hwang *et al.*, 2020). Interestingly, one study has bypassed the PGCLC stage and differentiated PSCs into spermatogonial stem cells and subsequently haploid cells, albeit with low efficiency (Xu *et al.*, 2020).

## Setting the stage for iPSCD-gametogenesis

For novel stem cell-based ARTs to make it to the clinic, it is ethically required that they undergo evaluation and meet certain quality standards before, during, and after their clinical application (Mousaei Ghasroldasht *et al.*, 2022). Still, evaluation frameworks can be difficult to find, and regulations can vary substantially between jurisdictions (Health Council of the Netherlands: Committee on In Vitro Fertilization, 1998; Pennings *et al.*, 2007a). In private sectors, the clinical implementation of innovative ARTs is furthermore frequently characterized by *trial-and-error* approaches (Dondorp and de Wert, 2011), with new reproductive techniques being introduced haphazardly and, once they appear to produce healthy offspring, quickly adopted as standard fertility treatments that can be offered to aspiring parents (Mulder *et al.*, 2018).

To prevent the clinical introduction of human SCD-gametes from occurring haphazardly, the International Society for Stem Cell Research (ISSCR, 2021) recommends prohibiting reproductive applications at present but also recognizes that prohibitions could be lifted in the future, if reproductive applications are ‘proven safe and remaining ethical issues are resolved’ (Clark *et al.*, 2021). Those anticipating the clinical introduction of SCD-gametes should be mindful of these and other relevant directives (de Wert *et al.*, 2014), reflecting critically on the feasibility of the technology and how it would balance against issues that include—but are not limited to—safety and effectiveness.

The aim of the following sections is to encourage that reflection by highlighting the breadth of ethical, legal, and societal issues involved in the potential clinical introduction of iPSCD-gametes. The first section discusses issues emerging from preclinical studies with non-human animals and/or human cells, which must be conducted before clinical trials can occur (Stage 1). Issues during clinical trials emerge from research studies conducted with human subjects before the availability of the technology on the market (Stage 2) and are discussed in the second section. The third section focuses on issues after clinical trials, referring to (studies on) the impact of the technology after it has become available on the market (Stage 3).

### Stage 1: Before clinical trials

Generating human iPSCD-gametes raises practical, ethical, legal, and societal hurdles. Firstly, the reprogramming method used and expanded culture required to introduce stemness in somatic cells of origin is currently associated with the increased occurrence of unintended or ‘off-target’ (epi)mutations (Chang *et al.*, 2021; Poetsch *et al.*, 2022). Moreover, the co-culture step currently required to induce PGCLCs into sex-specific pathways might be a limiting and sensitive factor, especially if it involves the retrieval and use of human fetal tissue acquired from elective abortions (Shorr, 2019; Brumbaugh *et al.*, 2023). Third, while recent patenting trends seem to show that ‘the iPSC field is gradually shifting toward applied innovation, disease modeling, and treatment’ (Lyu *et al.*, 2024), it remains unclear how patenting-related costs may condition the clinical introduction of SCD-gametes. Intellectual property issues ‘could come at the expense of robust competition, widespread innovation, and equitable access, creating a social divide between reproductive haves and have-nots’ (Cyranoski *et al.*, 2023). Intellectual property choices made at early, preclinical stages must thus be carefully considered as these can ultimately promote or undermine the major promises of iPSCD-gametes for potential beneficiaries: equitable and inclusive reproduction (Le Goff *et al.*, 2024).

Once human iPSCD-gametes can be generated, they must be compared to their *in vivo* counterparts for safety and efficacy assessment. Pups generated from mouse SCD-gametes (Hayashi *et al.*, 2011; Hikabe *et al.*, 2016) can provide a starting point for assessing the health of offspring (Mulder *et al.*, 2018; Serrano *et al.*, 2021). But to take a step further, insights are needed from parallel research with other (primate) animals, human gametes, and human embryos; typically, in that order (Jans *et al.*, 2018). These forms of research are not only technically challenging but also require careful ethical, legal, and societal consideration due to their historically greater sensitivity. For research with human gametes, the main concern is the scarce and burdensome donation of human oocytes (Pennings *et al.*, 2007b; Levens and DeCherney, 2008), although this may be partly alleviated if methods to mature oocytes *ex vivo* become more efficient in the future (Del Valle and Chuva de Sousa Lopes, 2023). For research with non-human primates (Arnason, 2018; Harvey and Kaiser, 2023) and human embryos (Robertson, 1986), the main concern stems from the commonly shared intuition that they are somehow similar to (if not already) human persons that, therefore, enjoy special (if not equal) status. Differences in terms of how to interpret this special status resulted in notably different national regulations that may hinder—or impede—the advancement of iPSCD-gametes. In jurisdictions where the creation of research embryos is banned, for example—including those that ratified the Oviedo Convention (Council of Europe, 1997)—researchers are prevented from conducting the parallel studies required to assess the quality of embryos generated from iPSCD-gametes. By extension, prohibitions on non-reproductive (i.e. research or therapeutic) cloning would also have to be contextually considered (Lisker, 2003; Segers *et al.*, 2019), as these could place additional restrictions on using iPSCD-gametes for fertilization in certain jurisdictions. Inconsistencies in cross-border regulatory frameworks have also been found to possibly exacerbate ethical and societal concerns in similar fields of research (Lensink *et al.*, 2021; Pereira Daoud *et al.*, 2024). It is worth exploring empirically whether the same can be expected in relation to research with iPSCD-gametes, for which national regulations or concrete frameworks are still lacking.

### Stage 2: During clinical trials

Once iPSCD-gametes can be generated and sufficiently assessed preclinically, the next hurdle is determining what conditions must be met to conduct clinical trials, and how these conditions translate to specific trial phases. In the context of clinical trials with experimental ARTs, two groups of participants are involved: those consenting to undergoing trial (aspiring parents), and those possibly resulting from it (future offspring). This adds issues to the normative agenda regarding clinical trials. To ensure that the interests of all participants are protected and not subordinate to those of science and society (Nordberg *et al.*, 2020), specific provisions are set out in international declarations, such as the Declaration of Helsinki (WMA, 2014) and the Universal Declaration on Bioethics and Human Rights (United Nations Educational, Scientific and Cultural Organization (UNESCO) 2005). Moreover, before each trial phase, the protocol is reviewed by an ethics review committee to assess whether the study contributes to new scientific insights, involves a proper methodology, proves non-inferiority to standard alternatives, ensures a responsible informed consent process, et cetera.

These conditions are often elaborated in international guidelines or national laws and policies but appropriate oversight and regulation for iPSCD-gametes remains lacking (National Academies of Sciences, Engineering, and Medicine, 2023). It is also unclear whether and how contemporary legal frameworks would apply to them (Hendriks *et al.*, 2015), potentially raising questions about standardization and arbitrariness in clinical trials (Yarborough, 2021). In the European Union, for example, the Clinical Trials Regulation (CTR) (European Parliament and Council, 2014) plays an important role regarding medical research involving human subjects. The scope of the CTR is determined by whether clinical trials involve a medicinal product. It is questionable whether iPSCD-gametes would qualify as medicinal products. However, this cannot be ruled out since clinical trials with different types of stem cells have been assessed under the CTR before (Bellino *et al.*, 2023). If clinical trials with iPSCD-gametes are subject to the CTR, it is important to consider that this regulation prohibits clinical trials that result in modifications to the subject’s germline.

While regulation would be welcomed, risks are ultimately inevitable; especially in first-in-human (FIH) trials, where preclinical research limitations can expose different research populations to varying degrees of harm (Dresser, 2009). Even once enough preclinical evidence is gathered, the full reproductive potential of iPSCD-gametes might only be known once the product of their *in vitro* fertilization is transferred to a receptive human uterus, putting at least those undergoing clinical pregnancy and those intended to be born from it at risk of serious harm—if not also future generations. This risk could be physical, psychological, or both and vary considerably across trials, for example, due to biological (e.g. mutations) or procedural (e.g. opposite sex gametogenesis) differences in generating the human iPSCD-gametes. Addressing questions of safety in trials with iPSCD-gametes will thus ultimately have to go beyond evidence-based risk assessments and be more readily understood as ‘a matter of proportionality’ (Jans *et al.*, 2020b): can the benefits of the technology outweigh its burdens? A main concern in this regard is that using iPSCD-gametes reproductively might seriously burden ‘the physical welfare of the resulting offspring (and possibly also that of following generations)’ (Segers *et al.*, 2017). What this welfare should minimally amount to remains difficult to define (Pennings *et al.*, 2007a), making it more challenging to weigh potential burdens against the aspired (Hendriks *et al.*, 2015; Le Goff *et al.*, 2024) yet contested (Cutas *et al.*, 2014; Mertes, 2014b) benefit of genetic parenthood.

Interestingly, empirical research indicates that potential beneficiaries of iPSCD-gametes might draw the line at risks ‘comparable to those of natural conception or IVF’ (Le Goff *et al.*, 2024). These findings raise immediate questions. Is conducting trials with iPSCD-gametes feasible, also in view of legal and ethical standards? Careful decision-making and well-informed and free consent of those undergoing the procedure are crucial to sound practice (Lowenthal *et al.*, 2012). Yet few people might be willing to accept or able to understand the risks involved, also raising questions about the scope and extent of the information provision required for providing proper informed consent at trial stages (National Academies of Sciences, Engineering, and Medicine, 2023). Substantial monetary incentives might be needed to increase the pool of participants and soften any harm that the trials might inflict on them but also risk encouraging misconduct or exploitation (Dresser, 2009), possibly putting population groups already susceptible to financial, legal, or social disadvantages (e.g. people with uteruses; Disi *et al.*, 2022; Patel *et al.*, 2024) at additional risk. Moreover, they raise the empirical—and ethically relevant—question of whether (‘full’) genetic parenthood is as valued as it has thus far been assumed to be. If that were the case, would involuntarily childless people not have been eager to accept a greater degree of risk?

From a scarce resource perspective, it is also important to consider whom should be given access to trials with iPSCD-gametes first and on what basis. Some have argued that ‘fertility restoration in heterosexual couples (…) is, for several reasons, likely to be the most widely accepted application’ (Hendriks *et al.*, 2015). This prompts the question of whether and when the benefit of iPSCD-gametes for specifically *equitable* and *inclusive* genetic parenthood can be expected. Moreover, it should be questioned whose perspectives are considered when it is stated that certain applications are more ‘widely accepted’. Generalizing claims about benefits and burdens evoke questions about the authority of those making these claims, and about the normativity of what it means to earmark something as ‘widely accepted’ or ‘controversial’ in the first place.

### Stage 3: After clinical trials

Many of the foregoing issues can be expected once iPSCD-gametes become readily available in fertility clinics. Questions about the role and responsibility of individual clinicians, for example, emerge both during and after clinical trials. Clinicians are accountable for their medical professional actions and expected to provide care according to the principles of good medical practice, which are again often further specified in (supra)national protocols and guidelines (e.g. ESHRE; Dondorp and de Wert, 2011; de Wert *et al.*, 2018). At its core, the responsibility of clinicians is to put the interests of their patients first and, in fertility settings, this responsibility includes multiple patients, like the prospective parent(s), eventual surrogate(s), and future children. Fertility specialists should therefore refuse to collaborate on parental projects if there is a high risk of serious harm to future children (Pennings *et al.*, 2007a). This dual responsibility may go unmet in fertility treatments with human iPSCD-gametes. As previously discussed, the risks of conceiving children with human iPSCD-gametes are hard to quantify and can differ considerably between individuals. This limits clinical knowledge about possible adverse effects in particular cases, undermining the clinicians’ ability to inform patients and the patients’ ability to give informed consent, putting both in a vulnerable position.

Generating offspring from human iPSCD-gametes would require substantial follow-up studies, with their duration depending on foreseeable risks (Health Council of the Netherlands: Committee on In Vitro Fertilization, 1998; Pennings *et al.*, 2007a; Jans *et al.*, 2020a,b). Should certain congenital disorders only become apparent in the offspring of the children conceived from human iPSCD-gametes, then this may imply having to monitor multiple generations, making it important to devise practically and monetarily feasible long-term plans before FIH trials can be initiated (Pennings *et al.*, 2007a). To be equitable, these plans must accommodate and bridge ethically relevant differences between patients (e.g. by including surrogacy). Moreover, patients should be made aware that they have a moral responsibility to inform their children about the way they were conceived (Pennings *et al.*, 2007a). This is important because children should have the choice of opting out of follow-up studies once they become legally competent and because not knowing about one’s conception might negatively affect the offspring’s psychological and emotional well-being. The latter has been observed in children conceived from gamete donation (Blyth, 2012; Freeman and Golombok, 2012; Ilioi *et al.*, 2017), although it remains to be established whether similar effects could be expected in children conceived with human iPSCD-gametes since they would be genetically related to their parents (Mertes and Pennings, 2010). The responsibility of informing future offspring about the way they were conceived can be difficult to accommodate legally, however. For example, due to the parents’ right to a private life, as protected under the European Convention on Human Rights (Council of Europe, 1950).

As observed in families with children conceived from contemporary ARTs (Golombok, 2015), monitoring family dynamics might also be important. However, further research is needed to establish if and how ARTs factor in the psycho-emotional well-being of children and families thus conceived. In the specific context of children conceived from iPSCD-gametes, post-clinical monitoring studies should consider how the technique might affect contemporary conceptions of the family and genetic parenthood, including whether it reinforces or undermines the (presumed) relevance of genetic ties.

Finally, once successfully established in fertility clinics, human iPSCD-gametes might have an impact on other ARTs, as well. For example, because it may be used to replace ovulation induction and oocyte retrieval, lowering burdens for oocyte donors (Pennings, 2023), and increasing the number of oocytes and, consequently, embryos available for screening and selection in preimplantation genetic testing (PGT) techniques (Greely, 2016; Chapman, 2022). In that respect, the clinical use of human iPSCD-gametes might have more benefits than currently considered, but it might also raise additional concerns. In the context of PGT, for example, it has been suggested that human iPSCD-gametes could ‘offer a greater range of choice in selecting the characteristics of prospective children’ (Chapman, 2022). Similar slippery slopes can be conceived in relation to natural conception, more broadly. If the safety and efficacy of human iPSCD-gametes surpass that of ‘natural’ gametes, could that lead to the medicalization and stigmatization of ‘naturally’ conceived pregnancies? Those making such reflections cannot ignore, however, that ‘autonomous choice’ is structured by factors like social relationships, expectations, and intersectional conditions, and that ‘historical and contemporary examples of control over women’s (gestating) bodies indicate that control is exercised much more on the bodies of disadvantaged members of society, such as women of color, members of ethnic minorities, and disabled and poor women’ (Cavaliere, 2020, 79).

## Concluding remarks

Contrary to recent estimates (Cyranoski *et al.*, 2023; National Academies of Sciences, Engineering, and Medicine, 2023), this mini-review suggests that an evidence-based approach to the reproductive application of human iPSCD-gametes may take longer than a decade. This is in part because of the current technical limitations in generating human iPSCD-gametes but also, and more fundamentally, because of the many ethical, legal, and societal questions that their clinical application would raise in the (near) future.

The issues discussed in the context of Stage 1 (before clinical trials) suggest that, even once current limitations are overcome, gathering the evidence required to preclinically assess the safety of human iPSCD-gametes will be time-consuming and require parallel experiments with ethically, legally, and societally sensitive research materials, like non-human primates, human oocytes, and human embryos. The issues discussed in the context of Stage 2 (during clinical trials) suggest that, even when enough preclinical evidence can be provided, it might still take several years (if not decades) for iPSCD-gametes to transition through distinct trial phases, and that inevitable (and unforeseeable) variations in the quality of patient-specific cells (biological or social heterogeneity) are likely to further slowdown this process. Finally, the issues discussed in the context of Stage 3 (after clinical trials) suggest that, even once trials have been conducted, offering iPSCD-gametes in fertility clinics will still require addressing questions of accountability and monitoring, some of which might be difficult to formalize by law.

Some of these issues might be resolved or avoidable by partial IVG protocols, including protocols that would involve ‘autotransplanting precursors of [human iPSCD-]gametes into patients’ testicles or ovaries’ (Hendriks *et al.*, 2015). Partial protocols were not the focus of this review but may provide worthwhile reproductive routes that should be explored further. On the other hand, these routes might be less promising for those anticipating equitable and inclusive human reproduction, specifically, and it might be interesting to investigate if and how this presumed disadvantage could influence its development.

Concluding, what to expect when expecting the clinical introduction of human iPSCD-gametes? The findings of this mini-review cannot rule out the possibility of human iPSCD-gametes being introduced haphazardly in fertility clinics in the near future. Yet in view of them, it seems unlikely that responsible and *evidence-based* approaches can occur within the next decade and/or immediately promote equitable and inclusive reproduction. This may undermine some of the major societal forces driving the development of human iPSCD-gametes. More likely for the near future is that human iPSCD-gametes will be used in research; for example, as robust disease models to understand and treat patient-specific fertility problems, providing indirect yet useful insights for developing new or improving contemporary ARTs. To make well-informed and well-considered choices about the development and application of human iPSCD-gametes, it will be important to address the full breadth of technical, ethical, legal, and societal issues in both reproductive and research settings, underscoring the importance of early and ongoing transdisciplinary collaborations with a broad range of stakeholders (Carayannis and Campbell, 2009; Stilgoe *et al.*, 2013).

## Data Availability

No new data were generated or analyzed in support of this research.
